# Upper Gastrointestinal Hemorrhage: Validation of the Severity Score

**DOI:** 10.4021/gr540w

**Published:** 2013-05-03

**Authors:** Rangson Chaikitamnuaychok, Jayanton Patumanond

**Affiliations:** a Department of General Surgery, Kamphaeng Phet Hospital, Kamphaeng Phet, 62000, Thailand; b Clinical Epidemiology Unit, Faculty of Medicine, Chiang Mai University, Chiang Mai, 50200, Thailand

**Keywords:** Clinical prediction rules, Scoring system, Screening, Upper gastrointestinal bleeding, Upper gastrointestinal hemorrhage, Validation

## Abstract

**Background:**

A simple scoring system was developed earlier to classify patients presenting with upper gastrointestinal hemorrhage into mild, moderates and severe. To validate the derived simple UGIH severity scoring system to another set of data obtained from consecutive patients.

**Methods:**

The score was developed earlier from data of patients with UGIH in 2009 - 2010. The same scoring system was assigned to another set of data from patients of the following year. Classification of patients into 3 urgency levels reflecting their severity was compared. Performance similarity of the score in the two sets of data was tested with a chi-squared test for homogeneity. The ability of the score to discriminate mild patients from moderate/severe, and to discriminate severe patients from mild/moderate was identified and compared with analysis of area under the receiver operating characteristic curve (AuROC).

**Results:**

Patients from the validation data were similar to those from the development data in overall aspects. The severity of UGIH and the score distribution in the two sets were similar. The score successfully classified patients in the validation data into 3 severity levels similar to the development data (P = 0.381, chi-squared for homogeneity), and similarly discriminated mild patients from moderate/severe patients (P = 0.360, AuROC analysis), and similarly discriminated severe patients from mild/moderate patients (P = 0.589, AuROC analysis)

**Conclusion:**

The simple scoring scheme developed earlier to classify UGIH patients into 3 severity/urgency levels performed similarly in the validation data obtained from patients in the following year. Advantages of the scoring scheme should be tested when applied to patient care to assure clinical adoption into routine practice.

## Introduction

Acute upper gastrointestinal hemorrhage (UGIH) is one among the most common clinical presentations in emergency departments [1]. The cost of case management is also high [2, 3]. It was estimated that, in the United States, one admission of such patients costs many thousand $US with an overall expense of more than one billion dollars annually [4-6]. The costs of care could have been reduced by shifting case management from in-patient to out-patient care [7]. In achieving such goal, selective screening of patients must be sufficiently effective, so that selected patients can safely be managed as out-patients [7].

In the past, risk stratification algorithms have successfully been developed for many diseases or clinical conditions, such as community acquired pneumonia, chest pain, and febrile neutropenia [7]. For UGIH, clinical profiles and endoscopic criteria were applied to classify patients focusing on adverse clinical conditions [8-12]. The most popular clinical outcomes considered in several studies were re-bleeding, emergency surgery required, mortality [10, 11], repeated endoscopy, re-admission, length of stay, unplanned OPD visit, and costs of care [7].

The main purpose of classifying patients with UGIH is to select those with low risk of re-bleeding or death [13], in order to avoid unnecessary or overuse of diagnostic and/or therapeutic interventions. Such avoidance would highly reduce the total cost of case managements [7], without increasing the patient risks. At the same time, selective screening for patients with high risk would reassure patient admission into hospitals or intensive care units for close monitoring, so that timely procedures may be scheduled to avoid complications or deaths.

We previously developed and reported a simple scoring system to classify UGIH patients into 3 severity/urgency levels, based on clinical and laboratory profiles without endoscopic examinations [14]. Compared to previous research, our score included common clinical variables such as age > 60 years, systolic blood pressure < 100 mmHg, presence of co-morbidity, and cirrhosis [15-17]. However, some clinical profiles proposed in previous works were intentionally excluded, either due to their low prevalence or difficulty in assessment, such as presence of ascites, blood-colored nasogastric tube aspirates [16], requiring blood transfusion > 5 Unit [17], elevated prothrombin time [18], and erratic mental status [18].

The objective of the present study was to validate the scoring system previously described, with an independent set of data of similar patients at another period of time.

## Patients and Methods

### Patients

The medical files of patients who presented with upper gastrointestinal hemorrhage at Kamphaeng Phet Hospital, a university affiliated general hospital in the lower northern region of Thailand, were reviewed. The key ICD-10 used for computer search was K920-hematemesis, K921-melena, and K922-gastrointestinal hemorrhage unspecified. Based on The American College of Surgeons [19], the same criteria for upper gastrointestinal hemorrhage severity were also used in the present study.

### Development data

Patients in the development data were those registered in 2009 and 2010 (n = 984).

### Validation data

The validation data came from similar patients registered in 2011 (n = 423).

### Statistical methods

The baseline characteristics of the development data and the validation data were compared with exact probability tests and t-tests. Severity scores were assigned to the patients in both sets (Table 1). Under-estimated and over-estimated proportions of severity were calculated and compared with a chi-squared for homogeneity test. Performances of the score in the development and the validation data were calculated by the areas under the receiver operating curves (AuROC). The discriminative ability of the score was displayed by the probability curves for each of the severity levels.

**Table 1:** Score Assignment Scheme for Classifying UGIH Severity

Clinical characteristics	Criteria	Assigned score
Age (year)	< 60	0
	≥ 60	1
Pulse (/min)	< 100	0
	≥ 100	1
Systolic pressure (mmHg)	< 100	10.5
	≥ 100	0
Hemoglobin (g/dL)	< 10	6
	≥ 10	0
BUN (mg/dL)	≤ 35	0
	> 35	2
Cirrhosis	no	0
	yes	2
Hepatic failure	no	0
	yes	4.5

Modified from the original scoring chart (with permission).

## Results

Baseline characteristics of patients in the development and the validation data were similar in almost all clinical parameters, except for the presence of renal failure, which was higher in the validation data (7.0% vs 11.6%, P = 0.006). The distributions of severity levels in the two data were also similar (P = 0.408). However, the percentage of re-bleeding in the validation data was significantly lower (P = 0.016), while the percentage of death was significantly higher (P = 0.008) (Table 2).

**Table 2:** Clinical Characteristics of UGIH Patients in the Development and the Validation Data

Characteristics	Development (n = 984)		Validation (n = 423)		P-value
	mean	± SD	mean	± SD	
Demographics					
Male (n, %)	651	66.2	298	70.4	0.121
Age (year)	58.6	± 15.6	59.4	± 16.5	0.407
Mode of presentation (n, %)					
Hematemesis	455	46.2	183	43.3	0.321
Coffee ground vomiting	196	19.9	76	18.0	0.419
Hematochezia	65	6.6	33	7.8	0.425
Melena	574	58.3	227	53.7	0.113
Syncope	192	19.5	73	17.3	0.335
Hemodynamics					
Pulse (/min)	91.4	± 16.0	91.5	± 16.0	0.879
SBP (mmHg)	119.0	± 23.0	121.3	± 23.3	0.078
Biochemicals					
Hemoglobin (g/dL)	8.1	± 2.9	8.0	± 2.9	0.511
BUN (mg/dL)	33.6	± 21.3	35.1	± 23.0	0.223
Co-morbidities (n, %)					
Cirrhosis	141	14.3	55	13.0	0.557
Hepatic failure	8	0.8	8	1.9	0.099
Cardiac failure	9	0.9	3	0.7	0.999
Renal failure	69	7.0	49	11.6	0.006
Criterion-classified severity (n, %)					
Mild	241	24.5	99	23.4	0.408
Moderate	631	64.1	268	63.4	
Severe	112	11.4	56	13.2	
Clinical outcomes (n, %)					
Re-bleeding	64	6.5	14	3.3	0.016
Dead	20	2.0	20	4.7	0.008

SD: standard deviation.

The mean severity score in the validation data was non-significantly lower (9.1 ± 5.9 vs 8.5 ± 5.7, P = 0.064) and the percentage of “emergent” group in the validation data was non-statistically smaller (22.2% vs 16.5%, P = 0.082) (Table 3).

**Table 3:** Score-Derived UGIH Urgency Levels in the Development and the Validation Data

Score-classified urgency levels	Development (n = 984)		Validation (n = 423)		P-value
Mean score (± SD)	9.1	± 5.9	8.5	± 5.7	0.064
Urgency level (n, %)					
Non-urgent	188	19.1	84	19.9	0.082
Urgent	578	58.7	269	63.6	
Emergent	218	22.2	70	16.5	

SD: standard deviation.

Categorizing patients with the score into 3 urgency levels in the validation data yielded the following results. Patients scoring less than 4 (non-urgent) predicted “mild severity” correctly in 73.7% (73 in 99), with 1-level under-estimation in 10 cases (2.4%) and 2-level under-estimation in 1 case (0.2%), a total of 2.6% under-estimations.

Patients scoring between 4 and 16 (urgent) predicted “moderate severity” correctly in 84.7% (227 in 268), with under-estimation in 16 cases (3.8%), and over-estimation in 26 cases (6.1%).

Patients scoring above 16 (emergent) predicted “severe severity” correctly in 69.6% (39 in 56), with 1-level over-estimation in 31 cases (7.3%) and 2-level over-estimation in no cases, a total of 7.3% over-estimations (Table 4).

**Table 4:** Score-Classified Urgency, and Criterion-Classified UGIH Severity in the Validation Data

Score-classified urgency levels		Score range	Criterion-classified severity levels			Risk estimation validity		
			Mild (n = 99)	Moderate (n = 268)	Severe (n = 56)	Over	Correct	Under
Mean ± SD			2.9 ± 4.0	9.0 ± 4.1	16.1 ± 4.9			
IQR*			0 - 4	7 - 9	12.0 - 19.5			
Non-urgen	(n = 84)	< 4	73	10	1	-	17.3	2.6
Urgent	(n = 269)	4 - 16	26	227	16	6.1	53.7	3.8
Emergent	(n = 70)	> 16	0	31	39	7.3	9.2	-
					Total	13.4	80.2	6.4

*Inter-quartile range; SD: standard deviation.

An overall severity level specific agreement of 80.2% (vs 81.4%), with an overall 6.4% (vs 7.5%) under-estimation and an overall 13.4% (vs 11.1%) over-estimation were similar to the development data [14], and were still clinically acceptable.

**Figure 1:**
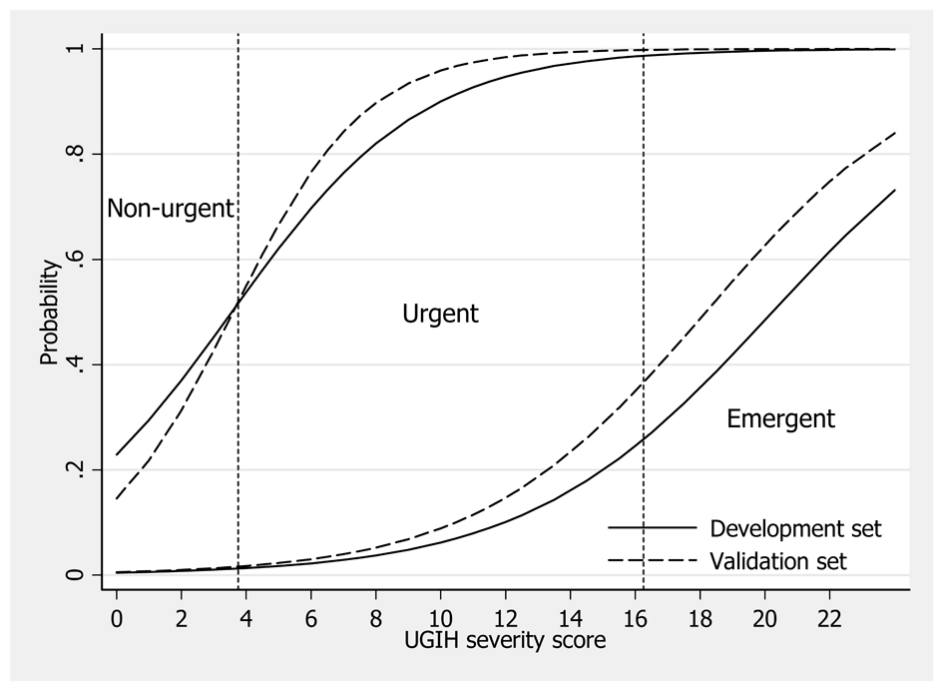
Score predicted probability of severity/urgency levels in the development data (solid lines) and the validation data (dash lines). Vertical dotted lines represent boundaries (cut-off points) for classifying patients into non-urgent, urgent and emergent groups.

The score also discriminated mild patients from moderate and severe patients in the two data similarly (AuROC = 84.11% and 86.92%, P = 0.360), and also discriminated severe patients from another 2 levels similarly (AuROC = 86.56% and 88.11%, P = 0.589), yielding similar prediction curves (Table 5 and Fig. 1).

**Table 5:** Discriminative Performance of the UGIH Severity Score in the Development and the Validation Data

Prediction/discrimination	Development (n = 984)		Validation (n = 423)		P-value
	AuROC (%)	95%CI	AuROC (%)	95%CI	
Mild vs rest	84.11	81.71 - 86.37	86.92	83.41 - 90.05	0.360
Severe vs rest	86.56	84.30 - 88.65	88.11	84.72 - 91.10	0.589

AuROC: area under receiver operating characteristic curve; CI: confidence interval.

## Discussion

Once a scoring system was developed, it is a common practice to validate it with another set of patients, preferable from an independent or external source. Existing scoring systems to screen and classify UGIH patients have also been validated to evaluate their performances. Validation of The Blatchford Score [20-22], indicated its advantageous performance in predicting requirement of interventions (such as surgery, blood transfusion, or endoscopy), over The Complete Rockall Score, or The Pre-endoscopic Rockall Score [20, 21]. The Blatchford Score successfully classified patients into “high” and “low” risk, before endoscopy was done [23]. The Rockall Score was more efficient in predicting mortality than in predicting re-bleeding [24-26].

The present scoring system classified UGIH patients in the validation data closed to the development data. Compared to the development data, the percents of correct prediction in the validation dataset were also similar (80.2% vs 81.4%). Although an overall under-estimation was less (6.4% vs 7.5%), while an over-estimation was more (13.4% vs 11.1%), these differences were not statistically different by a chi-squared test for homogeneity (P = 0.381), implying similar classifications. The fact that under-estimation was less and that over-estimation was more, also re-assured that the present scoring may be applied safely to future patients.

Screening and classifying UGIH patients has proved to reduce unnecessary medical costs, without increasing any adverse clinical risks to the patients. The cost of care was reduced from $3,940 to $340, when case managements were shifted from in-patients to out-patients [27]. Most screening procedures used non-sophisticated clinical parameters, as in The Blatchford Score [22, 23], or endoscopic parameters as in others [11, 12]. When validated, safe clinical outcomes had been achieved, with low re-bleeding rate and no surgical intervention was required in the group classified as “low risk”.

In clinical perspectives, the present severity scoring system is quite practical. The score components were also obtained from clinical and/or laboratory parameters, already available in routine practice. Besides, obtaining these parameters were not time-consuming, required no special or invasive procedures, and readily available in almost all levels of medical centers. As far as we are aware of, no contra-indications for applying these sorts of scoring systems were mentioned in the literature.

An application of the scoring system into routine practice may change UGIH case management in a positive direction. Non-urgent or “mild” patients may be managed more appropriately, no intervention may be required at all, and patients may be managed as out-patients safely, and endoscopy may be appointed later as elective cases. Urgent or “moderate” patients may be admitted into hospitals, for which endoscopy may be appointed during that admission (24 - 96 hours). For emergent or “severe” patients, they may be monitored closely, or admitted into an intensive care unit, for which endoscopy may be scheduled immediately, as soon as possible, or within 24 hours after the admission.

However, future research will be needed to evaluate when the score is put into routine clinical practice for UGIH case managements.

### Conclusions

Validation of the earlier derived UGIH severity score with patients of subsequent year showed similar performances. Correct severity level prediction was high, with clinically acceptable under- and over-estimation. The score may safely be applied into routine practice in medical centers and in patients with similar characteristics. Its performance should be re-evaluated when put into practice under routine patient care management.

## Abbreviations

UGIH: Upper gastrointestinal hemorrhage
OPD: Out-patient department
AuROC: Area under receiver operating characteristic curve

## References

[1] Palmer K (2007). Acute upper gastrointestinal haemorrhage. Br Med Bull.

[2] Longstreth GF (1995). Epidemiology of hospitalization for acute upper gastrointestinal hemorrhage: a population-based study. Am J Gastroenterol.

[3] Rockall TA, Logan RF, Devlin HB, Northfield TC (1995). Incidence of and mortality from acute upper gastrointestinal haemorrhage in the United Kingdom. Steering Committee and members of the National Audit of Acute Upper Gastrointestinal Haemorrhage. BMJ.

[4] Gralnek IM, Jensen DM, Kovacs TO, Jutabha R, Jensen ME, Cheng S, Gornbein J (1997). An economic analysis of patients with active arterial peptic ulcer hemorrhage treated with endoscopic heater probe, injection sclerosis, or surgery in a prospective, randomized trial. Gastrointest Endosc.

[5] Gralnek IM, Jensen DM, Gornbein J, Kovacs TO, Jutabha R, Freeman ML, King J (1998). Clinical and economic outcomes of individuals with severe peptic ulcer hemorrhage and nonbleeding visible vessel: an analysis of two prospective clinical trials. Am J Gastroenterol.

[6] Quirk DM, Barry MJ, Aserkoff B, Podolsky DK (1997). Physician specialty and variations in the cost of treating patients with acute upper gastrointestinal bleeding. Gastroenterology.

[7] Gralnek IM (2002). Outpatient management of "low-risk" nonvariceal upper GI hemorrhage. Are we ready to put evidence into practice?. Gastrointest Endosc.

[8] Longstreth GF, Feitelberg SP (1995). Outpatient care of selected patients with acute non-variceal upper gastrointestinal haemorrhage. Lancet.

[9] Longstreth GF, Feitelberg SP (1998). Successful outpatient management of acute upper gastrointestinal hemorrhage: use of practice guidelines in a large patient series. Gastrointest Endosc.

[10] Rockall TA, Logan RF, Devlin HB, Northfield TC (1995). Variation in outcome after acute upper gastrointestinal haemorrhage. The National Audit of Acute Upper Gastrointestinal Haemorrhage. Lancet.

[11] Rockall TA, Logan RF, Devlin HB, Northfield TC (1996). Risk assessment after acute upper gastrointestinal haemorrhage. Gut.

[12] Rockall TA, Logan RF, Devlin HB, Northfield TC (1996). Selection of patients for early discharge or outpatient care after acute upper gastrointestinal haemorrhage. National Audit of Acute Upper Gastrointestinal Haemorrhage. Lancet.

[13] Bordley DR, Mushlin AI, Dolan JG, Richardson WS, Barry M, Polio J, Griner PF (1985). Early clinical signs identify low-risk patients with acute upper gastrointestinal hemorrhage. JAMA.

[14] Chaikitamnuaychok R, Patumanond J (2012). Upper gastrointestinal hemorrhage: development of the severity score. Gastroenterology Res.

[15] Chaikitamnuaychok R, Patumanond J (2012). Clinical risk characteristics of upper gastrointestinal hemorrhage severity: a multivariable risk analysis. Gastroenterology Res.

[16] Corley DA, Stefan AM, Wolf M, Cook EF, Lee TH (1998). Early indicators of prognosis in upper gastrointestinal hemorrhage. Am J Gastroenterol.

[17] Yavorski RT, Wong RK, Maydonovitch C, Battin LS, Furnia A, Amundson DE (1995). Analysis of 3,294 cases of upper gastrointestinal bleeding in military medical facilities. Am J Gastroenterol.

[18] Kollef MH, O'Brien JD, Zuckerman GR, Shannon W (1997). BLEED: a classification tool to predict outcomes in patients with acute upper and lower gastrointestinal hemorrhage. Crit Care Med.

[19] Committee on Trauma, American College of Surgeons (2008). ATLS: Advanced trauma life support program for doctors.

[20] Stanley AJ, Ashley D, Dalton HR, Mowat C, Gaya DR, Thompson E, Warshow U (2009). Outpatient management of patients with low-risk upper-gastrointestinal haemorrhage: multicentre validation and prospective evaluation. Lancet.

[21] Schiefer M, Aquarius M, Leffers P, Stassen P, van Deursen C, Oostenbrug L, Jansen L (2012). Predictive validity of the Glasgow Blatchford Bleeding Score in an unselected emergency department population in continental Europe. Eur J Gastroenterol Hepatol.

[22] Chen IC, Hung MS, Chiu TF, Chen JC, Hsiao CT (2007). Risk scoring systems to predict need for clinical intervention for patients with nonvariceal upper gastrointestinal tract bleeding. Am J Emerg Med.

[23] Masaoka T, Suzuki H, Hori S, Aikawa N, Hibi T (2007). Blatchford scoring system is a useful scoring system for detecting patients with upper gastrointestinal bleeding who do not need endoscopic intervention. J Gastroenterol Hepatol.

[24] Sanders DS, Carter MJ, Goodchap RJ, Cross SS, Gleeson DC, Lobo AJ (2002). Prospective validation of the Rockall risk scoring system for upper GI hemorrhage in subgroups of patients with varices and peptic ulcers. Am J Gastroenterol.

[25] Enns RA, Gagnon YM, Barkun AN, Armstrong D, Gregor JC, Fedorak RN (2006). Validation of the Rockall scoring system for outcomes from non-variceal upper gastrointestinal bleeding in a Canadian setting. World J Gastroenterol.

[26] Vreeburg EM, Terwee CB, Snel P, Rauws EA, Bartelsman JF, Meulen JH, Tytgat GN (1999). Validation of the Rockall risk scoring system in upper gastrointestinal bleeding. Gut.

[27] Cipolletta L, Bianco MA, Rotondano G, Marmo R, Piscopo R (2002). Outpatient management for low-risk nonvariceal upper GI bleeding: a randomized controlled trial. Gastrointest Endosc.

